# A Small Platoon of Idiots Have Been Allowed to Flourish, but the Homunculus Has Not Been Erased from Memory

**DOI:** 10.5334/joc.106

**Published:** 2020-09-10

**Authors:** Cai S. Longman

**Affiliations:** 1 Psychology, School of Education and Social Sciences, University of the West of Scotland, Paisley, UK

**Keywords:** cognitive control, learning, attention, eye movements

## Abstract

Schmidt, Liefooghe and De Houwer’s (2020) PEP model is able to explain many empirical effects commonly reported in task switching experiments without invoking an executive control homunculus. However, their claim that they have erased the homunculus from memory may be a little premature. Although they have gone a long way in dissolving, deconstructing and fractionating the executive, there remain several empirical effects that are difficult to explain under PEP, some of which they openly discuss. In the present commentary, I have described some findings from my own research on spatial attention in task switching using eye-tracking that I think PEP would also struggle to model, but which can easily be explained by active control processes. I conclude that PEP still has some way to go before the homunculus can be altogether erased from memory.

Although Schmidt, Liefooghe and De Houwer’s (2020) PEP model has certainly gone some way toward answering Monsell and Driver’s (2000, p. 7) call to “dissolve, deconstruct, or fractionate the executive” and they have let at least four or five (though maybe not quite a hundred) “idiots flourish” (p. 7), I do not believe that they have succeeded in erasing the ever-elusive homunculus from memory. Schmidt and colleagues are clear about their intention to describe some of the “nuts and bolts” (p. 57) of task-set control in terms of the binding processes modelled by PEP (a goal I am sure Monsell & Driver would endorse). However, they also note some of the empirical effects commonly found in task switching experiments that PEP might struggle to model (e.g., strategic preparatory effects). Although the authors offer some avenues by which PEP might succeed in modelling these effects, they also acknowledge that they might be best explained by active control processes. I will report some other findings from my own investigations of spatial attention in task switching using eye-tracking that I think are also difficult to explain under PEP. Although I have to agree with Schmidt and colleagues’ view that binding effects might play a part in the studies I report here, I think their results merit further consideration.

Longman et al. (2014, 2016, 2017, see also Longman et al., 2013) recorded eye movements during the cue-target interval (CTI) in a series of task switching experiments where participants had to perform one of three number classification tasks. The target stimulus was three digits displayed at the corners of an invisible equilateral triangle. Each task was bound to a single location throughout the experiment (e.g., the odd/even task should always be performed on the uppermost digit) so that, given sufficient time, participants could use the preparation interval to fixate the task-relevant location.^1^ We found a behavioural switch cost that reduced with preparation, but remained significant at the longest CTI (1420 ms). Importantly, we also consistently found a delay in fixating the task-relevant digit on switch trials relative to repeats. This was in part explained by initial fixations on the task-irrelevant regions on switch trials but a strong and consistent delay was found even after controlling for these orienting ‘errors’. Nonetheless, fixations on the task-irrelevant digits on switch trials were much more likely to be on the digit associated with the task that had been performed on trial *n*-1 – an effect we referred to as ‘attentional inertia’. Both of these effects remained reliable at the longest CTI, but were largely limited to conditions where spatial attention was (re)oriented as part of a task-set and were modulated by the nature of the cues. In one experiment, the same number classification task was performed throughout the session, and the cue only indicated which digit to classify. Under these conditions, the delay in orienting and attentional inertia were eliminated at the longer CTIs. In another experiment, we manipulated the extent to which the cues indicated the relevant classification task or the relevant digit-location. Even when participants were required to switch between classification tasks, cues that explicitly indicated the relevant digit resulted in the elimination of both eye-tracking effects. Taken together, this pattern of results suggests that resetting attentional parameters of a task-set is a time-consuming process, and that it is difficult to overcome inertia in these parameters. That we found these effects only when spatial attention was (re)oriented as part of a task-set, or with cues that indicated the task is difficult to explain under PEP. According to this model, the priming effect of repeating the relevant stimulus location between trials should be equivalent whether the classification task switches or not and whether the cues indicate the to-be-performed task or the relevant digit.

In our 2017 paper, we gave participants ultimate control over the duration of the preparation interval by instructing them to maintain their gaze on the central task-cue until they were ready to perform the relevant task. The stimulus appeared as soon as they shifted their gaze away from the cue. We partitioned the data into quartiles based on the duration of the self-paced preparation interval and found that although the (residual) switch cost and the delay in orienting remained reliable at the two longest CTIs (mean durations over participants: 671 ms and 1134 ms), participants were able to eliminate attentional inertia. That is, they did not preferentially fixate the digit associated with the task performed on trial *n*-1 on switch trials. Indeed, the proportion of fixations on either of the task-irrelevant digits in this experiment was very low in all but the shortest CTI (mean duration = 466 ms). Taken together with the findings from our earlier studies that consistently found attentional inertia at longer CTIs, this pattern of results seems to indicate that participants were using the self-paced preparation interval to actively prepare for the upcoming task and monitor their readiness to perform it. It is not easy to explain either of these processes under PEP without deferring to an executive control homunculus to do the jobs of active preparation (rapidly overcoming attentional inertia) and monitoring the preparation process for signs that it had been achieved.

In summary, I am impressed by how many of the common empirical findings in the task switching literature Schmidt and colleagues (2020) were able to model without invoking an executive. However, I am not altogether convinced that they have erased the homunculus from memory once and for all. Although I appreciate the work they have done so far in allowing a small platoon of idiots to flourish (Monsell & Driver, 2000), I look forward to seeing how much more of the executive they can dissolve, deconstruct, or fractionate in the future.

## Footnotes

Note that cue and stimulus repetitions were controlled in these experiments, but decision and response repetitions were not. Because each task was consistently mapped to a single digit-location throughout the experiment, it was also not possible to control for task-location bindings and the between-trial benefits that such a feature would no-doubt allow.
